# Wogonin protects glomerular podocytes by targeting Bcl-2-mediated autophagy and apoptosis in diabetic kidney disease

**DOI:** 10.1038/s41401-021-00721-5

**Published:** 2021-07-12

**Authors:** Xue-qi Liu, Ling Jiang, Yuan-yuan Li, Yue-bo Huang, Xue-ru Hu, Wei Zhu, Xian Wang, Yong-gui Wu, Xiao-ming Meng, Xiang-ming Qi

**Affiliations:** 1 Department of Nephropathy, The First Affiliated Hospital of Anhui Medical University, Hefei, 230022 China; 2 Center for Scientific Research of Anhui Medical University, Hefei, 230022 China; 3 The Key Laboratory of Major Autoimmune Diseases, Anhui Institute of Innovative Drugs, School of Pharmacy, Anhui Medical University, The Key Laboratory of Anti inflammatory and Immune Medicines, Ministry of Education, Hefei, 230022 China

**Keywords:** diabetic kidney disease, wogonin, podocytes, autophagy, apoptosis, Bcl-2

## Abstract

Diabetic kidney disease (DKD) is one of the microvascular complications of diabetes mellitus and a major cause of end-stage renal disease with limited treatment options. Wogonin is a flavonoid derived from the root of *Scutellaria baicalensis* Georgi, which has shown a potent renoprotective effect. But the mechanisms of action in DKD are not fully elucidated. In this study, we investigated the effects of wogonin on glomerular podocytes in DKD using mouse podocyte clone 5 (MPC5) cells and diabetic mice model. MPC5 cells were treated with high glucose (30 mM). We showed that wogonin (4, 8, 16 μM) dose-dependently alleviated high glucose (HG)-induced MPC5 cell damage, accompanied by increased expression of WT-1, nephrin, and podocin proteins, and decreased expression of TNF-α, MCP-1, IL-1β as well as phosphorylated p65. Furthermore, wogonin treatment significantly inhibited HG-induced apoptosis in MPC5 cells. Wogonin reversed HG-suppressed autophagy in MPC5 cells, evidenced by increased ATG7, LC3-II, and Beclin-1 protein, and decreased p62 protein. We demonstrated that wogonin directly bound to Bcl-2 in MPC5 cells. In HG-treated MPC5 cells, knockdown of Bcl-2 abolished the beneficial effects of wogonin, whereas overexpression of Bcl-2 mimicked the protective effects of wogonin. Interestingly, we found that the expression of Bcl-2 was significantly decreased in biopsy renal tissue of diabetic nephropathy patients. In vivo experiments were conducted in STZ-induced diabetic mice, which were administered wogonin (10, 20, 40 mg · kg^−1^ · d^−1^, i.g.) every other day for 12 weeks. We showed that wogonin administration significantly alleviated albuminuria, histopathological lesions, and p65 NF-κB-mediated renal inflammatory response. Wogonin administration dose-dependently inhibited podocyte apoptosis and promoted podocyte autophagy in STZ-induced diabetic mice. This study for the first time demonstrates a novel action of wogonin in mitigating glomerulopathy and podocytes injury by regulating Bcl-2-mediated crosstalk between autophagy and apoptosis. Wogonin may be a potential therapeutic drug against DKD.

## Introduction

Diabetic kidney disease (DKD) remains a growing health concern and is characterized by chronic inflammation, hemodynamic changes, and metabolic dysfunction [1, 2]. The early pathological changes in DKD mainly include podocyte injury, shed, and apoptosis, while the surviving podocytes show compensatory hypertrophy and podocyte fusion [3, 4]. Podocyte injury induces proteinuria and leads to the continuous progression of DKD [5]. As podocytes have limited repair and regeneration ability, the degree of podocyte damage is the main factor determining the prognosis of DKD [6]. At present, prevention and control of DKD in clinical practice mainly includes early diagnosis, improved control of blood glucose, and the use of angiotensin-converting enzyme inhibitors or angiotensin receptor blockades [7]. Although these treatments delay the progression of DKD to a certain extent, they cannot prevent progression to end-stage renal disease. Therefore, the specific molecular mechanism and effective treatment of DKD need to be further explored.

Chinese herbal medicines are widely used for the treatment of diabetes and its complications [8–10]. Among these, wogonin is a flavonoid derived from the root of *Scutellaria baicalensis* Georgi, which has potent renoprotective effects [11, 12]. It has significant pharmacological actions, such as anti-inflammatory, anti-apoptotic, anti-oxidative, and cell cycle regulatory effects, in a variety of diseases [13]. We previously reported that wogonin administration in rodent models reduced RIPK1-mediated necroptosis following cisplatin-induced acute kidney injury [8]; however, its underlying mechanism in the early stages of DKD needs to be evaluated.

Autophagy and apoptosis play important roles in the development and cellular homeostasis. The autophagy level of podocytes is significantly higher than that of other renal intrinsic cells [14]. Studies have shown that podocyte autophagy substrate protein p62 is greatly accumulated and LC3-II expression is downregulated in the DKD model, indicating the inhibition of autophagy activity [15]. Apoptosis is an important factor in the occurrence of DKD. It has been found that various factors, such as glycolipid toxicity, angiotensin, and glycosylation end products, induce podocyte apoptosis through different pro-apoptotic pathways, leading to the occurrence of DKD [16]. Autophagy and apoptosis may be triggered by common upstream signals, resulting in combined autophagy and apoptosis or may be mutually exclusive [17].

The present study aimed to evaluate the effect of wogonin on glomerular podocytes in DKD in vitro and in vivo using mouse podocyte clone 5 (MPC5) cells and diabetic mice models. We further investigated the mechanism of wogonin. In the present study, we found that wogonin, used as a single agent, is sufficient to significantly reduce proteinuria and decrease podocyte injury. Its mechanism of action was found to be through targeted binding to Bcl-2, a well-characterized apoptosis guard that represents a molecular link between autophagy and apoptosis [18]. Our findings clearly showed that the increased activation of Bcl-2 by wogonin results in the mitigation of Bax-mediated apoptosis and promotion of Beclin-1-mediated autophagy in diabetic kidneys. Therefore, wogonin is expected to become a promising drug for the treatment of DKD.

## Materials and methods

### Chemicals and reagents

Wogonin was obtained from Aladdin Biology Technology Institute (W101155, CAS 632-85-9, Shanghai, China). STZ, *D*-glucose, and *D*-mannitol were procured from Sigma-Aldrich (Sigma, St. Louis, MO, USA). Antibodies specific for WT-1, nephrin, podocin, Bax, Bcl-2, cleaved caspase-3, and β-actin were acquired from Abcam Biotechnology (Abcam, Cambridge, MA, USA). Anti-ATG7, anti-p-p65, anti-p62, anti-p65, and anti-Beclin-1 antibodies were acquired from Cell Signaling Technology (Danvers, MA, USA). Periodic acid–Schiff (PAS) kit was obtained from Jiancheng Biotechnology Institute (Nanjing, China).

### Animals model and experimental design

Male C57BL/6 J mice (6‒8 weeks) were purchased from the Experimental Animal Centre of Anhui Medical University (Hefei, China). The present study protocol was approved by the Ethics Committee of Animal Research of Anhui Medical University (Hefei, China) and conformed to the NIH Guide for the Care and Use of Laboratory Animals. The experimental animals were kept in cages at 12 h light/dark cycle with free access to food and water in a laboratory with controlled temperature (22 ± 2°C) and humidity (60%). The mice were randomly divided into six groups: normal control (NC), NC + wogonin (40 mg/kg), STZ group, STZ + wogonin groups (10 mg/kg, 20 mg/kg, 40 mg/kg). After adaptable feeding for 7 days, 50 mg/kg STZ (dissolved in 0.1 M citrate buffer, pH 4.5) was intraperitoneally administered after 12 h of food deprivation daily for 5 consecutive days to generate DM mice [7]. Diabetes mice were defined by fasting blood glucose >250 mg/dL 2 weeks after the first STZ injection. The mice in NC+ wogonin and STZ+ wogonin groups were administered by intragastrical gavage with wogonin every other day for 12 weeks. NC and STZ groups were given intragastrically with the same amount of saline. At the end of 12 weeks, 24 h urine was collected from all mice using the metabolic cage. The mice were anesthetized by inhalation of 5% isoflurane and collected blood in the state of fasting. All experimental subjects were euthanized after anesthesia.

### Biochemical analyses

The 12 h fasting blood glucose levels in mice were measured every 2 weeks according to the Accu-Chek glucose Meters (Roche Diagnostic, Inc, Basel, Switzerland) recommended by the Diabetes Complications Animal Model Consortium. The kidney weight and bodyweight of each experimental animal were collected. Urine albumin was determined using an ELISA kit (Abcam, Cambridge, MA, USA) and urine protein (UP) was tested by ELISA Kit (Jianglai Biotechnology Co., Ltd, Shanghai, China) according to the product protocols. We collected mice blood samples in the fasted state by heart punctures. Collected blood was centrifuged for testing of blood urea nitrogen (BUN) and serum creatinine (Cr) (Beyotime, Haimen, China).

### Kidney histology

After the kidney was removed, it was fixed with 4% paraformaldehyde for 16 h. The renal tissues were embedded in paraffin and then cut into 4 μm sections. After dewaxing with xylene, the kidney structure was identified. The sections were stained using PAS and examined under a microscope (Zeiss Spot, Carl Zeiss, Gottingen, Germany) at ×400 magnification. Finally, the mesangial expansion index and tubular-interstitial injury index of renal tissue were evaluated from 10 randomly selected fields.

### Immunohistochemistry

The kidney sections were heated in a microwave at 95°C for 20 min and incubated with 3% hydrogen peroxide. The paraffin tissue sections were treated with anti-p62, anti-TNF-α, anti-IL-1β, anti-MCP-1, anti-Bcl-2, anti-nephrin, and anti-WT-1 antibodies for 24 h at 4°C, followed with the secondary antibody for 30 min at 37°C. After staining with DAB for 5 min, the slides were observed under a microscope (Zeiss Spot, Carl Zeiss, Gottingen, Germany).

### Cell culture

The MPC5 cells were procured from the Cell Bank of the Chinese Academic of Sciences (Shanghai, China) and grown in low-glucose Dulbecco’s Modified Eagle’s Medium (DMEM) (Gibco, San Diego, CA, USA) cultivated with 10% fetal bovine serum (Gibco, San Diego, CA, USA). MPC5 cells were propagated at 33°C and treated with interferon (IFN-γ; 10 U/mL). Next, cells were differentiated without IFN-γ at 37°C for 14 days. For further study, the MPC5 cells were stimulated with high glucose (30 mM glucose) and mannitol (24.5 mM mannitol + 5.5 mM glucose) containing 1% FBS with or without incubation of wogonin for 24 h.

### Transmission electron microscopy

MPC5 cells and renal tissues were treated with 2.5% glutaraldehyde and 1% osmic acid and fixed at 4°C for 3 days. The samples were added with 1% uranyl acetate and embedded in epoxy resin (EPON). Polymerized in gelatin capsules at 60°C for 48 h. The sections were observed under a transmission electron microscope (Hitachi, Japan).

### MTT (thiazole blue colorimetry) assay

MPC5 cells were cultivated in 96-well plates and incubated with a set of concentrations of wogonin, and cells were simultaneously incubated with high glucose for 24 h. Finally, MTT solution (5 mg/mL) was added to plates and incubated for 4–6 h. The optical density (OD) of each well was determined by the microplate reader (Multiskan MK3, Thermo Scientific, Waltham, MA, USA) at 550 nm wavelength.

### Bcl-2 overexpression and knockdown in MPC5 cells by shRNA transfections

Bcl-2 shRNA (Hanbio, Shanghai, China) was transfected into MPC5 cells mixed with Lipofectamine^TM^ 3000 reagent (Invitrogen, Carlsbad, CA, USA). The negative scrambled shRNA was used accordingly. First, the mixture of Lipofectamine^TM^ 3000 and shRNA were incubated for 20 min at room temperature in the dark, and then applied to the cells. After cultivation for 6 h, the cells were cultured in low-glucose DMEM with 10% FBS. Vector and lentivirus Bcl-2 (Hanbio, Shanghai, China) (multiplicity of infection, MOI = 10) were added into the MPC5 cells. Polybrene (10 μg/mL; Solarbio, Shanghai, China) was mixed with the growth medium to increase transfection efficiency. After 48 h cultivation, 2 μg/mL puromycin (Solarbio, Shanghai, China) was added to the growth medium. Stably transfected cells were screened for 2 weeks. The surviving cells were considered stable Bcl-2-overexpressing MPC5 cells. Western blot and real-time PCR were used to detect the knockdown and overexpression efficiency of Bcl-2.

### RNA isolation and real-time PCR

Renal tissue and cellular RNA were extracted by adding TRIZOL lysis buffer (Invitrogen, Carlsbad, CA, USA), and its concentration and purity were assessed using NanoDrop2000 spectrophotometer (Thermo Scientific, Waltham, MA, USA). After the sample quality was unified, the sample volume was calculated based on the concentration. The RNA sample was reverse-transcripted to cDNA and the primers for mRNA was designed. CFX96 real-time PCR system (Bio-Rad, Hercules, CA, USA) was used to perform on Real-time PCR assay by SYBR Premix Ex Taq™ II (Takara, Japan). PCR amplification conditions were as follows: running for over 40 cycles of denaturation at 95°C for 20 s, annealing at 58°C for 20 s, and elongation at 72°C for 20 s. The primers used in this assay are shown in Table 1.

**Table 1:** Sequences of primers

Genes	Forward (5′–3′)	Reverse (5′–3′)
Mouse Bcl-2	CCTGTGGATGACTGAGTACCTG	AGCCAGGAGAAATCAAACAGAGG
Mouse TNF-α	CATCTTCTCAAAATTCGAGTGACAA	TGGGAGTAGACAAGGTACAACCC
Mouse IL-1β	CTTTGAAGTTGACGGACCC	TGAGTGATACTGCCTGCCTG
Mouse MCP-1	CTTCTGGGCCTGCTGTTCA	CCAGCCTACTCATTGGGATCA
Mouse β-actin	GCGTGACATCAAAGAGAAGC	GCGTGACATCAAAGAGAAGC

### Western blot

Radio-immunoprecipitation assay buffer (Beyotime, Haimen, China) was used to extract the fragments of renal tissue and cellular proteins. The extracted protein concentrations were quantified by the bicinchoninic acid reagent (Beyotime, Haimen, China). The samples (40 μg) were separated via 10% or 15% sodium dodecyl sulfate-polyacrylamide gel electrophoresis and were transferred to nitrocellulose membranes. The membranes were sealed with 5% skimmed milk for 1 h, washed with phosphate-buffered saline (PBS) three times, and incubated with applicable primary antibodies overnight. They were then incubated with the secondary antibody (Rockland Immunochemicals Inc., PA, USA) for 30 min. The band intensities were exposed using a chemiluminescence system (Bio-Rad, Hercules, CA, USA). Image J software (NIH, Bethesda, MD, USA) was used to quantitatively analyze the protein bands after normalization with β-actin.

### Immunofluorescence assay

MPC5 cells were grown on glass coverslips, fixed with acetone at 37°C for 10 min, and then blocked with 10% bovine serum albumin (Beyotime, Haimen, China) at 37°C for 10 min. They were incubated with the primary antibody overnight, washed three times with PBS, and finally incubated with goat anti-rabbit IgG-rhodamine (Bioss, Beijing, China) antibody for 1 h in the dark at 37°C. The cells were counterstained with DAPI to stain the nuclei and imaged using an inverted fluorescence microscope (Zeiss Spot, Carl Zeiss, Gottingen, Germany).

### Molecular docking

Molecular docking was performed to gain an insight into the potential interactions between wogonin and Bcl-2. Discovery Studio (DS) 2017 R2 software (The Scripps Research Institute, La Jolla, CA, USA) was employed in this research. The structure of wogonin was optimized by minimizing protocol. The X-ray crystal structure of Bcl-2 (PDB code: 6QGH) was downloaded from the RCSB Protein Data Bank. Bcl-2 was prepared by preparing a protein protocol. CDOCKER module was applied to perform molecular docking. Set other parameters as default.

### Flow cytometry

Cells were washed with PBS three times and harvested by trypsin digestion. After incubation with 10 µL Annexin V-FITC and 5 µL propidium iodide in the dark, the cells were analyzed using a BD FACSVerse flow cytometer (BD Bioscience, San Jose, CA, USA). The rate of apoptosis was expressed by Annexin V^+^/PI^−^ (early apoptosis) and Annexin V^+^/PI^+^ (late apoptosis) status.

### TUNEL assay

MPC5 cell apoptosis was detected by TUNEL assay using the TUNEL Apoptosis Assay Kit (Beyotime, Haimen, China). The cells were incubated in terminal deoxynucleotidyl transferase (TDT)-mediated dUTP-biotin nick end-labeling (TUNEL). The TUNEL positive nuclei were observed by optical microscope (Leica DM4P, Shanghai, China).

### Cellular thermal shift assay (CETSA)

Cellular proteins were extracted from MPC5 cells with or without wogonin for 24 h. Total protein was regulated to the same concentrations using the Protein Assay Kit (Beyotime, Haimen, China). The proteins were distributed in PCR tubes and denatured at different temperatures (37–65°C) for 8 min on a PCR cycler (Bio-Rad, Hercules, CA, USA). After cellular protein was centrifuged, the proteins were then frozen and thawed three times with liquid nitrogen, and a Western blot was used to detect the proteins in the supernatant.

### Immunoprecipitation (IP) assay

Cells were suspended in standard NP-40 IP buffer (Sigma, St. Louis, MO, USA) and the proteins were incubated in NP-40 IP buffer at 4°C. The mixture was combined with anti-Bcl-2 antibody 3 h later and then precipitated with Protein G Sepharose beads followed by overnight incubation at 4°C. The beads were washed three times with 1 mL IP buffer and finally eluted. The immune complexes were then subjected to Western blot for determination of protein expression.

### Human kidney tissue

This clinical study has been approved by the Biomedical Ethics Committee of Anhui Medical University (Hefei, China) (#20170064) and followed the principles of the Declaration of Helsinki. Normal kidney samples (*n* = 6) were obtained from renal cancer patients who underwent routine nephrectomy at the First Affiliated Hospital of Anhui Medical University. Type 1 DM patients (*n* = 6) were selected. Inclusion criteria: (1) diagnosed with Type 1 diabetes over 10 years; (2) renal biopsy diagnosed as diabetic nephropathy; (3) 24 h urine albumin protein >300 mg/24 h; (4) no obvious symptoms of pyrexia and infection, no cancer, no autoimmune diseases. Fresh renal tissues were fixed with formalin for 24 h and embedded in paraffin until use.

### Statistical analysis

All experiments were independently conducted in triplicate. Data are expressed as mean ± SD and the difference between groups was considered statistically significant at *P* < 0.05. Statistical significance between groups was determined by the unpaired *t* test or ANOVA followed by Tukey’s post hoc analysis using GraphPad Prism 5 software (GraphPad Software Inc., San Diego, CA, USA).

## Results

### Wogonin alleviates HG-induced cellular damage in MPC5 cells

**Fig. 1:**
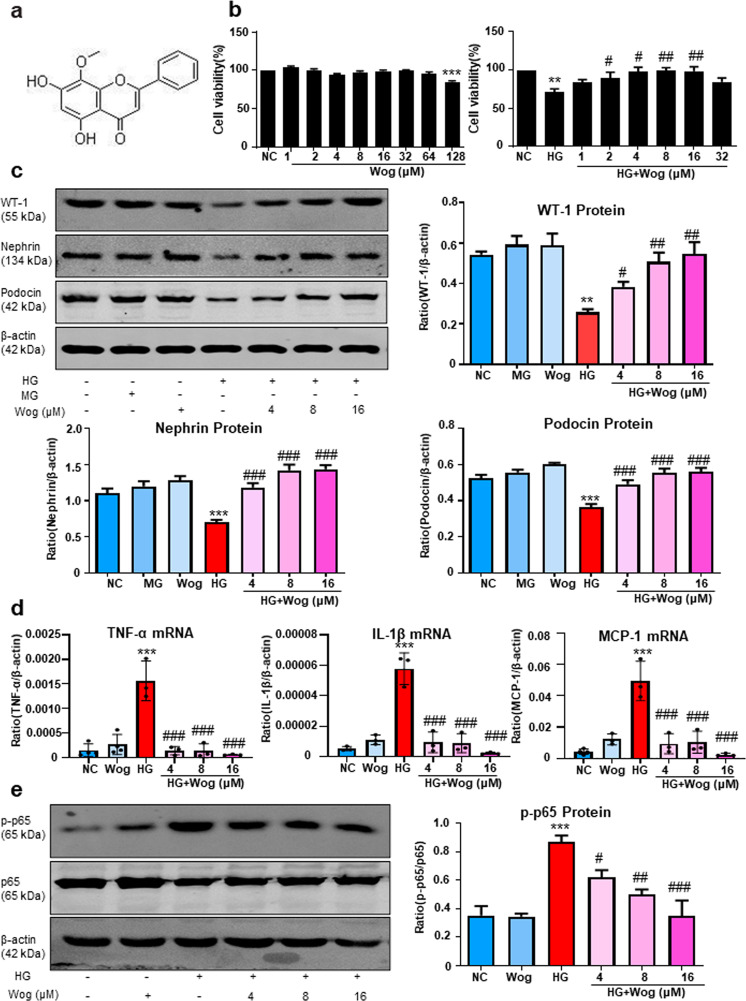
Effect of wogonin on HG-treated MPC5 cells’ viability and inhibition on HG-induced inflammation. **a** The molecular structural formula of wogonin. **b** MTT assay to determine the effect of wogonin on viability of MPC5 cells and HG-treated MPC5 cells. **c** Western blot of WT-1, nephrin, and podocin in MPC5 cells. **d** Real-time PCR analysis of TNF-α, MCP-1, and IL-1β expression in MPC5 cells. **e** Western blot analysis of phosphorylated p65 (p-p65) in MPC5 cells. Results represent mean ± SEM of three independent experiments. *P < 0.05, **P < 0.01, ***P < 0.001 vs NC. ^#^*P* < 0.05, ^##^*P* < 0.01, ^###^*P* < 0.001 vs HG. *HG* high glucose, *MG* 5.5 mM glucose plus 24.5 mM mannitol, *NC* normal control, *Wog* wogonin

The molecular structure of wogonin was shown in Fig. 1a. We used the MTT assay to assess the cytotoxic effect of wogonin and determine the adequate concentration used in the subsequent experiments (Fig. 1b). Wogonin showed a minimal effect on MPC5 cell viability at concentrations <64 μM. In fact, the best effect to restore the cell viability by wogonin was observed at concentrations of 4, 8, and 16 μM. Furthermore, the expression of podocyte-specific markers WT-1 and slit diaphragm proteins (SDs) including nephrin, and podocin were markedly rescued by wogonin treatment in a dose-dependent manner (Fig. 1c).

### Wogonin reduces the HG-induced inflammation response

Real-time PCR results showed the protective effect of wogonin against inflammatory response, as evidenced by decreased levels of inflammatory cytokines (IL-1β, MCP-1, and TNF-α) that were upregulated by high glucose (HG) stimulation (Fig. 1d). Moreover, we found that wogonin reduced the HG-induced phosphorylation of p65 in a dose-dependent manner (Fig. 1e).

### Wogonin promotes HG-suppressed autophagy in MPC5 cells

**Fig. 2:**
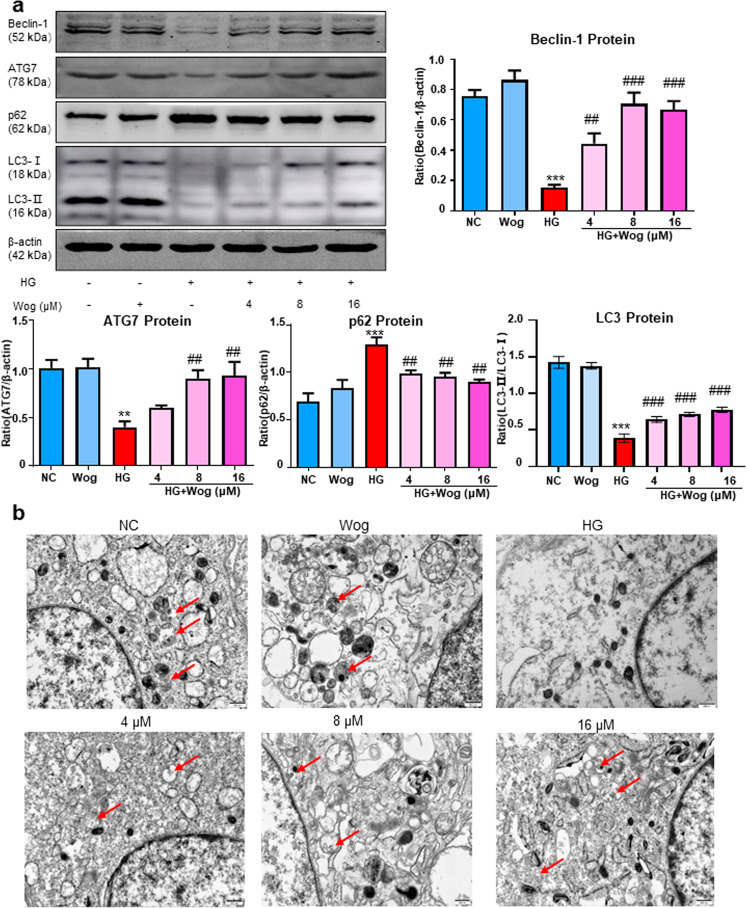
Wogonin promotes HG-induced autophagy disorders. **b** Western blot analysis of beclin-1, ATG7, LC3, and p62 in MPC5 cells. **b** Representative transmission electron microscopy images of autophagosomes in MPC5 cells, the arrows indicate autophagosomes. Scale bar = 500 nm. Results represent mean ± SEM of three independent experiments. **P < 0.01, ***P < 0.001 vs NC. ^##^*P* < 0.01, ^###^*P* < 0.001 vs HG. *HG* high glucose, *NC* normal control, *Wog* wogonin

Western blot analysis showed that wogonin treatment increased the ATG7, LC3-II, and Beclin-1 protein levels that were downregulated due to HG stimulation. Furthermore, the protein level of p62 was decreased with the treatment of wogonin (Fig. 2a). The transmission electron microscopy results also showed that wogonin significantly promoted autophagy in HG-treated MPC5 cells. The number of typical autophagosomes with double membranes was increased in podocytes with wogonin treatment (Fig. 2b).

### Wogonin alleviates HG-induced apoptosis in MPC5 cells

**Fig. 3:**
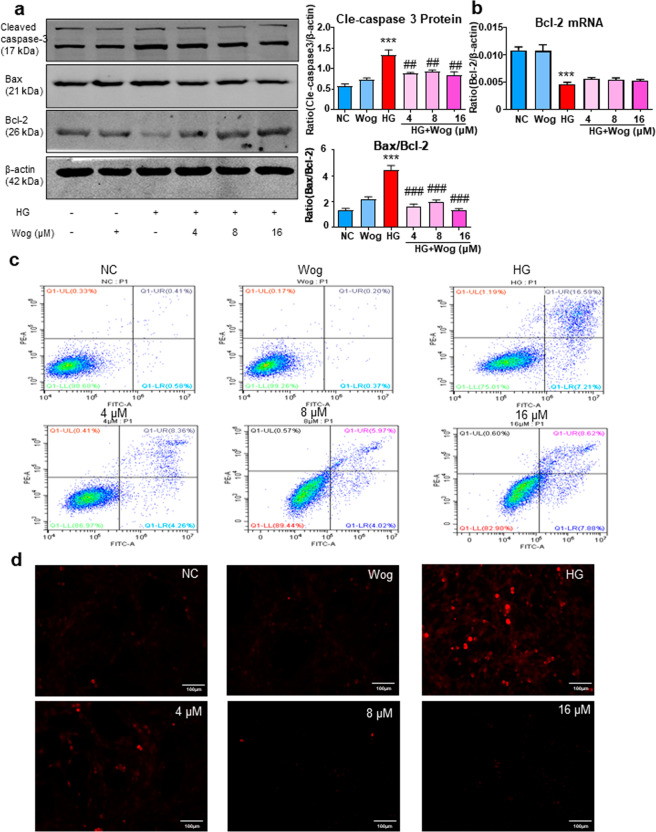
Wogonin inhibits HG-induced apoptosis. **a** Western blot analysis of cleaved caspase-3, Bax, and Bcl-2 in MPC5 cells. **b** Real-time PCR analysis of Bcl-2 in MPC5 cells. **c** Flow cytometry analysis of PI/Annexin V-stained MPC5 cells. **d** TUNEL assay for MPC5 cells. Scale bar = 50 μm. Results represent mean ± SEM of three independent experiments. ***P < 0.001 vs NC. ^##^*P* < 0.01, ^###^*P* < 0.001 vs HG. Abbreviations: *HG* high glucose, *NC* normal control, *Wog* wogonin

Wogonin treatment caused a decrease in the protein levels of cleaved caspase-3, and Bax and an increase in Bcl-2 protein levels in MPC5 cells (Fig. 3a). Interestingly, we found that the treatment of MPC5 cells with wogonin induced the protein, but not mRNA expression of Bcl-2 (Fig. 3b). Therefore, these data suggested that wogonin enhanced the expression of Bcl-2 by inhibiting the degradation of Bcl-2 in MPC5 cells rather than increasing the production of Bcl-2. Consistent with the above results, flow cytometry data of PI/Annexin V-stained MPC5 cells showed that wogonin reduced HG-induced cell apoptosis (Fig. 3c). Moreover, TUNEL staining showed that wogonin reduced the level of apoptosis (Fig. 3d).

### Wogonin target prediction

**Fig. 4:**
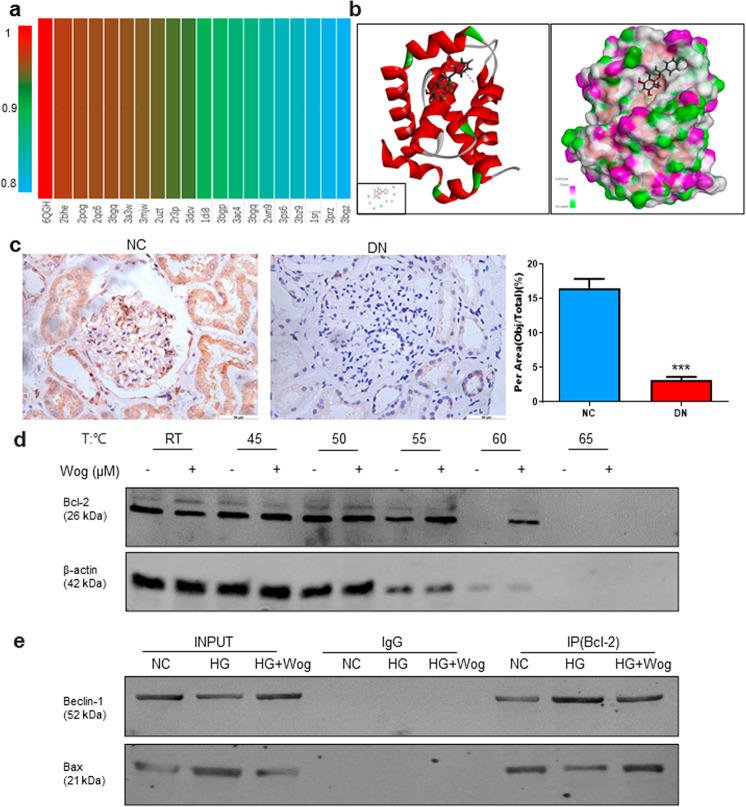
Prediction of wogonin molecular targets. **a** Profiling of the predicted protein targets of wogonin using Discovery Studio 2017 software. **b** Molecular docking of wogonin binding to Bcl-2 crystal structure. **c** Immunohistochemistry analysis of Bcl-2 in human kidney. Scale bar = 50 μm. Data represent the mean ± SEM for 6 humans. **d** CETSA analysis in MPC5 cells. **e** Immunoprecipitation assay. Results represent mean ± SEM of three independent experiments. ***P < 0.001 vs NC. *HG* high glucose, *NC* normal control, *Wog* wogonin

The target prediction of wogonin was carried out using DS 2017 software. As shown in Fig. 4a, the range of binding strength of wogonin to its potential target is shown in red to blue. The fit values represented the scores of the hypothetical targets, and the top ten disease-related targets were shown in Table 2. Among these targets, Bcl-2, a critical apoptosis-related protein in kidney disease, showed a high score of 0.9933 and appeared at a higher frequency, as shown in the mapping.

**Table 2:** Top ten putative protein targets of wogonin predicted using Discovery Studio 2017

Rank	PDB ID	Putative target	Fit value
1	6QGH	B-cell lymphoma-2	0.9933
2	2BHE	Cell division protein kinase 2	0.9868
3	2POG	Estrogen receptor	0.9864
4	2QC6	Casein kinase II subunit alpha	0.9846
5	3BGQ	Proto-oncogene serine/threonine-protein kinase Pim-1	0.9830
6	3A3W	Phosphotriesterase	0.9799
7	3MJW	Phosphatidylinositol-4,5-bisphosphate 3-kinase catalytic subunit gamma isoform	0.9762
8	2UZT	cAMP-dependent protein kinase, alpha-catalytic subunit	0.9727
9	2R3P	Cell division protein kinase 2	0.9660
10	3DCV	Proto-oncogene serine/threonine-protein kinase Pim-1	0.9644

### Wogonin binds directly to Bcl-2 in MPC5 cells

In this article, to study the binding posture between wogonin and Bcl-2 (PDB ID:6QGH), we used molecular docking. The 2D and 3D images showed that wogonin formed three pi-alkyl bonds with VAL76, ILE62, and ILE72. In addition, wogonin and Bcl-2 formed five van der Waals interactions with THR88, ILE89, PHE92, ILE67, and ARG65, which contributed to the binding affinity. The highest docking score detected by CDOCKER interaction energy was 42.1329 kcal/mol (Fig. 4b). Importantly, we found that Bcl-2 expression levels were decreased in renal tissues of patients with diabetic nephropathy (Fig. 4c). The target engagement between wogonin and Bcl-2 protein was performed by CETSA. Results indicated that the denaturation temperature of Bcl-2 differed by 50–65°C with or without wogonin. In MPC5 cells treated with wogonin, the thermal stability of Bcl-2 was significantly improved, showing that wogonin binds to Bcl-2 protein directly (Fig. 4d).

Moreover, bioinformatics prediction pointed out that wogonin may target Bcl-2. The results of the IP assay further confirmed the co-precipitation of Bcl-2 with Beclin-1 and Bax in MPC5 cells. Wogonin stimulation promoted Bcl-2–Bax-binding power and decreased Bcl-2–Beclin-1-binding ability, indicating that autophagy was increased, and apoptosis was reduced in the cells (Fig. 4e).

### Wogonin attenuates HG-induced cell apoptosis and promotes autophagy through Bcl-2-dependent mechanisms

**Fig. 5:**
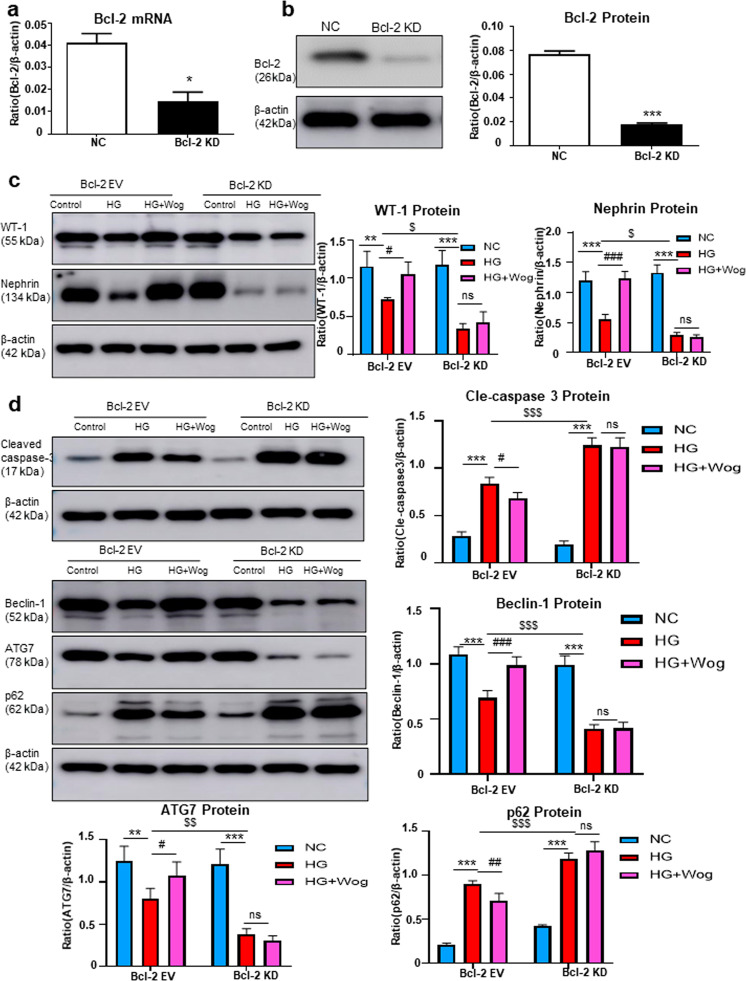
Wogonin fails to reduce the HG-induced cell injury, inflammatory response and promotes autophagy in Bcl-2-silenced MPC5 cells. **a** Real-time PCR analysis of Bcl-2 in MPC5 cells. **b** Western blot analysis of Bcl-2 in MPC5 cells. **c** Western blot analysis of WT-1 and nephrin in MPC5 cells. **d** Western blot analysis of cleaved caspase-3, Beclin-1, ATG7, and p62 in MPC5 cells. Results represent means ± SEM for three independent experiments. *P < 0.05, **P < 0.01, ***P < 0.001 vs NC. ^#^*P* < 0.05, ^##^*P* < 0.01, ^###^*P* < 0.001 vs HG. ^*$*^*P* < 0.05, ^*$$*^*P* < 0.01, ^*$$$*^*P* < 0.001 vs Bcl-2 EV group. *HG* high glucose, *Wog* wogonin, *EV* empty vector, *KD* Knockdown, *NC* normal control

First, we knocked down Bcl-2 in MPC5 cells using Bcl-2 shRNA (Fig. 5a, b). Inhibition of Bcl-2 expression could significantly increase cell apoptosis and suppress autophagy. After Bcl-2 was inhibited, wogonin was unable to further increase the expression of WT-1 and nephrin (Fig. 5c). This showed that in the absence of Bcl-2, wogonin was unable to exert its cellular protective role, suggesting that its function was through targeting Bcl-2 (Fig. 5d).

**Fig. 6:**
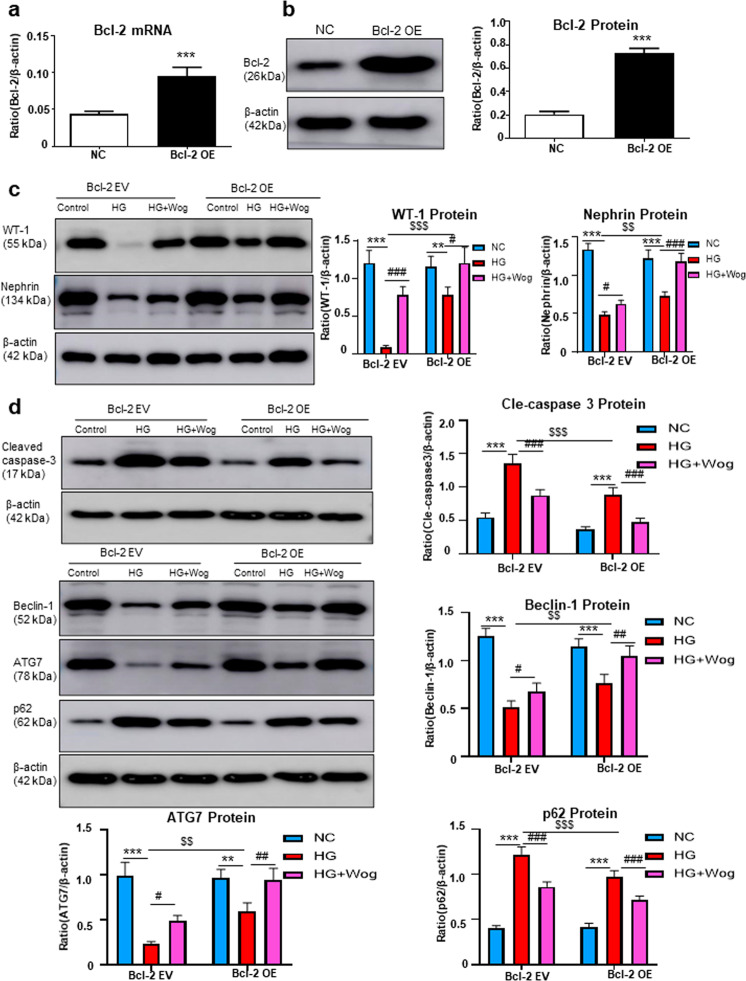
Bcl-2 overexpression and treatment with wogonin have similar cellular protective roles. **a** Real-time PCR analysis of Bcl-2 in MPC5 cells. **b** Western blot analysis of Bcl-2 in MPC5 cells. **c** Western blot analysis of WT-1 and nephrin in MPC5 cells. **d** Western blot analysis of cleaved caspase-3, Beclin-1, ATG7, and p62 in MPC5 cells. Results represent means ± SEM for three independent experiments. **P < 0.01, ***P < 0.001 vs NC. ^#^*P* < 0.05, ^##^*P* < 0.01, ^###^*P* < 0.001 vs HG. ^*$$*^*P* < 0.01, ^*$$$*^*P* < 0.001 vs Bcl-2-OE group. *HG* high glucose, *Wog* wogonin, *EV* empty vector, *OE* overexpression, *NC* normal control

Next, we overexpressed Bcl-2 using a lentivirus (Fig. 6a, b) and observed that Bcl-2 overexpression and wogonin treatment played similar cellular protective roles by increasing WT-1 and nephrin levels, suppressing cell apoptosis, and promoting autophagy. Moreover, in Bcl-2 overexpressing cells, the wogonin treatment showed a superposition effect to reduce cell injury (Fig. 6c, d).

### Wogonin alleviates physical and biochemical markers and pathology in STZ-induced type 1 diabetes mice model

**Fig. 7:**
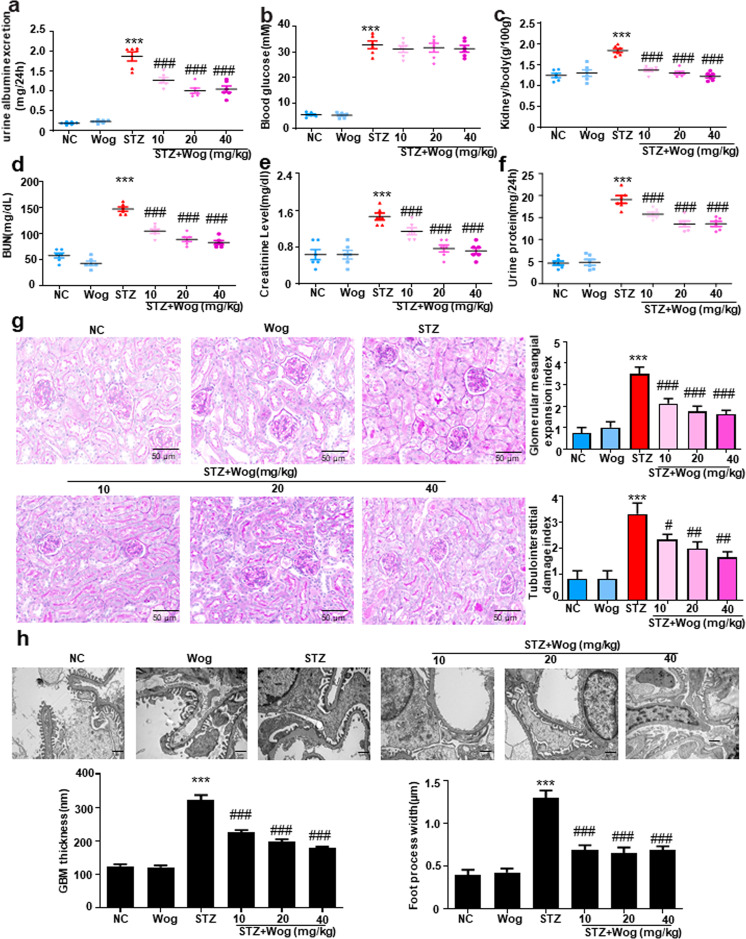
Physical and biochemical markers and histopathology of type 1 diabetic mice. **a** Analysis of urine albumin excretion. **b** Fasting blood glucose in different groups. **c** Kidney/body weight. **d** serum BUN assay. **e** serum Cr assay. **f** Twenty-four hours urinary protein quantitation. **g** Histological observations of kidney sections stained with periodic acid–Schiff (PAS) from different groups treated with or without wogonin. Scale bar = 50 μm. **h** Representative transmission electron microscopy images of podocyte foot processes and glomerular basement membrane. Scale bar = 1 μm. Results represent mean ± SEM for 6–8 mice. ***P < 0.001 vs NC. ^#^*P* < 0.05, ^##^*P* < 0.01, ^###^*P* < 0.001 vs STZ. *NC* normal control, *Wog* wogonin, *STZ* streptozotocin

For purpose of detecting whether wogonin could protect the kidneys in type 1 diabetic mice, diabetic mice were administered by intragastrical gavage with wogonin (10, 20, 40 mg/kg body weight). Diabetic mice showed polydipsia, polyuria, and polyphagia accompanied by significant weight loss, hair color messy and dirty, and slow movement. But these symptoms were improved significantly in mice treated with wogonin. At 12 weeks, diabetic mice showed significantly increased albuminuria that decreased after wogonin treatment (Fig. 7a). Treatment with wogonin could not alleviate the blood glucose levels that were markedly higher in STZ-induced DM group (Fig. 7b). Moreover, the kidney/weight ratio, BUN, Cr and 24-h UP were remarkably decreased after wogonin treatment (Fig. 7c–f). PAS staining showed that wogonin mitigated the degree of mesangial dilatation and tubulointerstitial injury in the DM group (Fig. 7g). The transmission electron microscopy results also showed that wogonin significantly alleviated renal basement membrane thickening and foot process fusion in type 1 diabetic mice (Fig. 7h).

### Wogonin protects glomerular podocytes in STZ-induced type 1 diabetes mice model

**Fig. 8:**
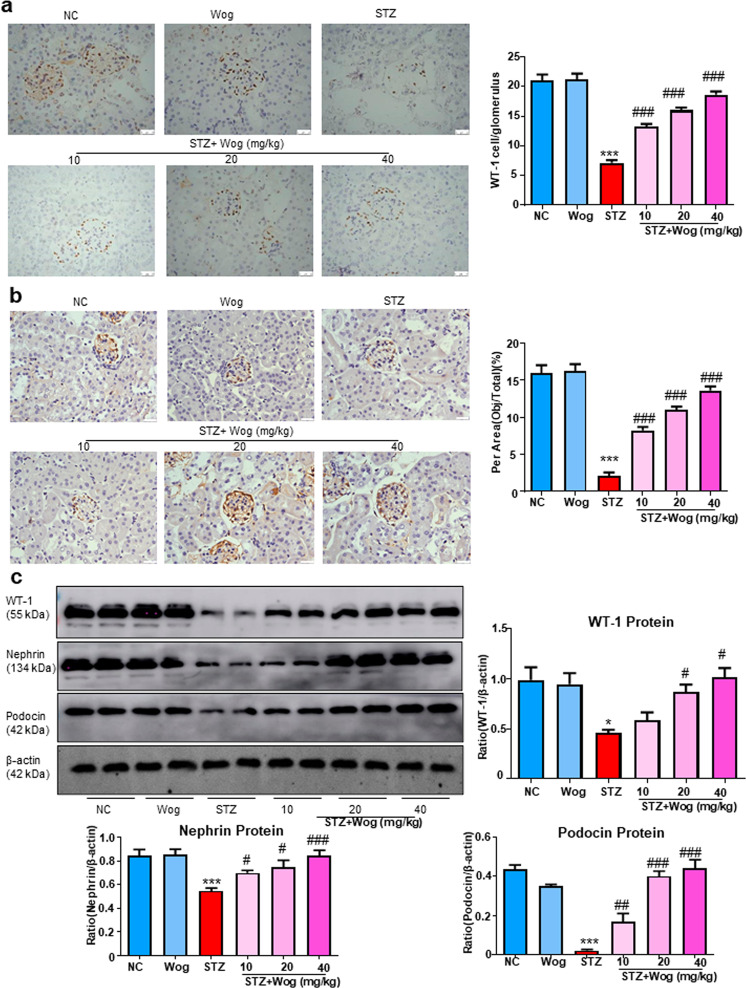
Wogonin attenuates glomerular podocytes injury in type 1 diabetic mice. **a** Immunohistochemistry analysis of WT-1 in mice kidney. Scale bar = 50 μm. **b** Immunohistochemistry analysis of nephrin in mice kidney. Scale bar = 50 μm. **c** Western blot analysis of WT-1, nephrin, and podocin in mice kidney. Data represent the mean ± SEM for 6–8 mice. *P < 0.05, ***P < 0.001 vs NC. ^#^*P* < 0.05, ^###^*P* < 0.001 vs STZ. *NC* normal control, *Wog* wogonin, *STZ* streptozotocin

Consistent with in vitro findings where wogonin could prevent HG-induced podocyte injury, WT-1, and nephrin in glomerular podocytes from diabetic mice were reversed by wogonin treatment. We observed that the podocyte-specific marker and SDs levels significantly decreased as quantified by staining WT-1 and nephrin in the diabetic group. These changes in diabetic mice were attenuated to different degrees by wogonin treatment and revealed similar histomorphology to that observed in non-diabetic controls (Fig. 8a, b). Further, Western blot results showed that wogonin increased expressions of WT-1, nephrin, and podocin in type 1 diabetic mice (Fig. 8c).

### Wogonin protects STZ-induced type 1 diabetes mice model by ameliorating inflammatory response

**Fig. 9:**
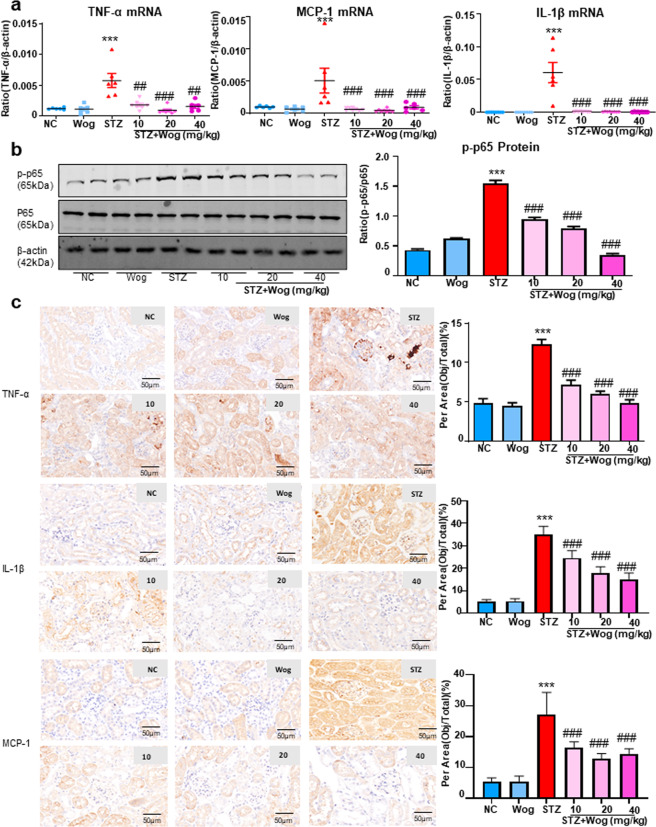
Wogonin attenuates renal inflammation in type 1 diabetic mice. **a** Real-time PCR analysis of inflammation (TNF-α, MCP-1, IL-1β) indices in mice kidney samples. **b** Western blot analysis of phosphorylated p65 (p-p65) in mice kidney tissues. **c** Immunohistochemistry analysis of TNF-α, MCP-1, IL-1β in mice kidney tissues. Scale bar = 50 μm. Data represent the mean ± SEM for 6–8 mice. ***P < 0.001 vs NC. ^##^*P* < 0.01, ^###^*P* < 0.001 vs STZ. *NC* normal control, *Wog* wogonin, *STZ* streptozotocin

Wogonin significantly reduced the levels of TNF-α, MCP-1, and IL-1β, as determined by real-time PCR (Fig. 9a). Further, results of Western blot revealed that wogonin suppressed phosphorylation of p65 in type 1 diabetic mice (Fig. 9b). Immunohistochemical data of IL-1β, MCP-1, and TNF-α also demonstrated the anti-inflammatory effect of wogonin in type 1 diabetic mice (Fig. 9c).

### Wogonin promotes podocyte autophagy in STZ-induced type 1 diabetes mice model

**Fig. 10:**
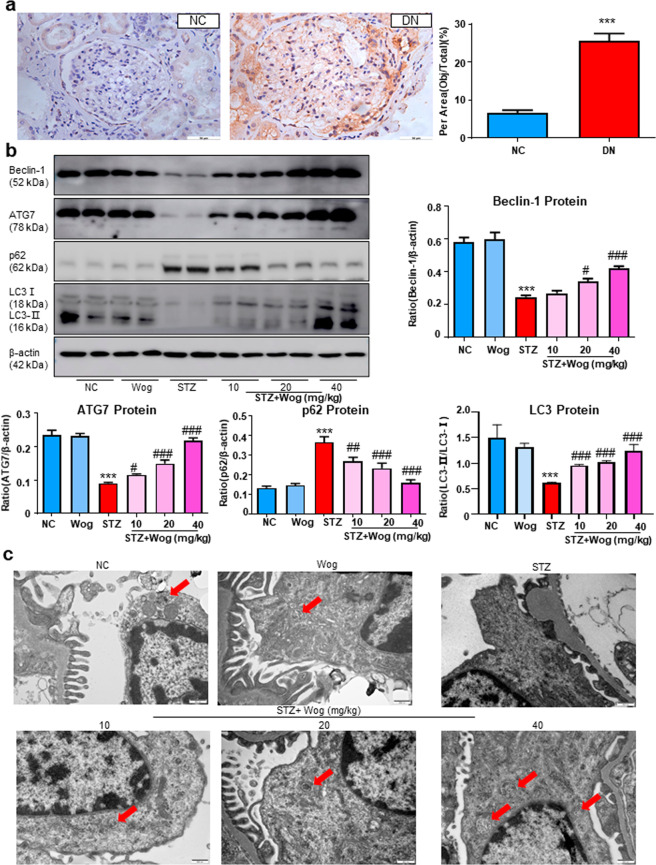
Wogonin attenuates renal autophagy disorder in type 1 diabetic mice. **a** Immunohistochemistry analysis of p62 in human kidney tissues. Scale bar = 50 μm. Data represent the mean ± SEM for six humans. **b** Western blot analysis of Beclin-1, ATG7, p62, and LC3 in mice kidney tissues. **c** Representative transmission electron microscopy images of autophagosomes in mice kidney tissues, the arrows indicate autophagosomes. Scale bar = 500 nm. Data represent the mean ± SEM for 6–8 mice. ***P < 0.001 vs NC. ^#^*P* < 0.05, ^##^*P* < 0.01, ^###^*P* < 0.001 vs STZ. *NC* normal control, *Wog* wogonin, *STZ* streptozotocin

Interestingly, we first found that the expression level of p62 was significantly increased in renal tissues of diabetic nephropathy patients (Fig. 10a). We next tested the effect of wogonin on podocyte autophagy in diabetic mice. Western blot analysis showed decreased autophagy-related proteins and concurrently increased p62 in kidney tissues of diabetic mice (Fig. 10b). Notably, the changes in Beclin-1, ATG7, LC3-II, and p62 levels in the renal tissue of diabetic mice were partially but significantly reversed by wogonin treatment. In addition, the number of typical autophagosomes with double membranes and the morphology of podocytes were observed by transmission electron microscopy. Reduced autophagosomes were found in the diabetic mice group, which increased after wogonin treatment (Fig. 10c).

### Wogonin ameliorates apoptosis in STZ-induced type 1 diabetes mice model

**Fig. 11:**
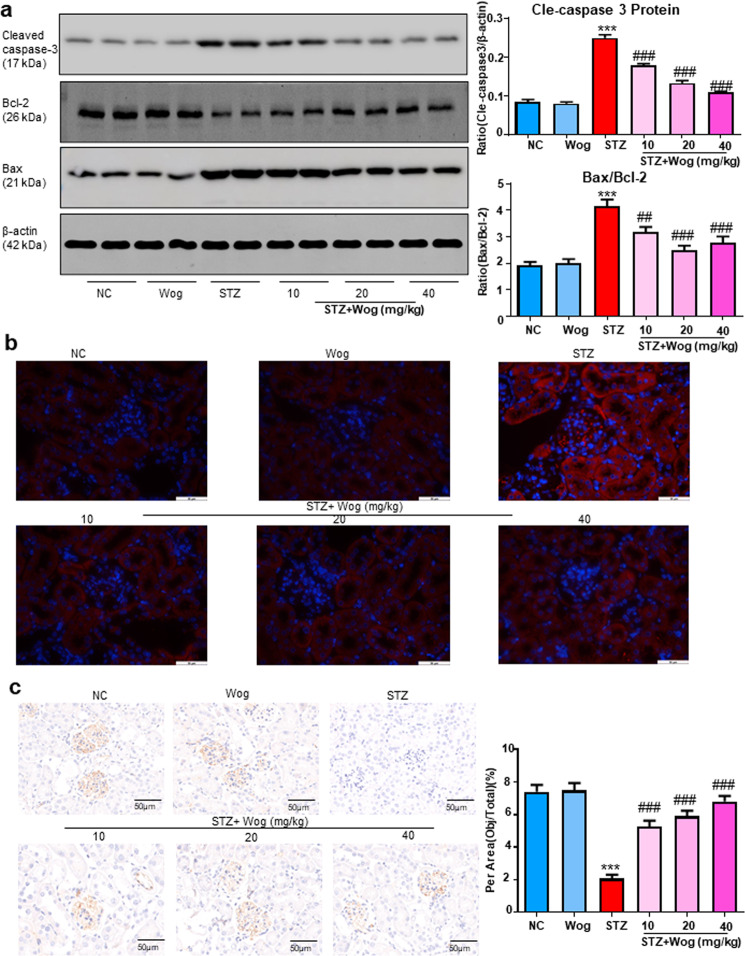
Wogonin attenuates cell death in type 1 diabetic mice. **a** Western blot analysis of cleaved caspase-3, Bax, and Bcl-2 in mice kidney tissues. **b** TUNEL assay in mice kidney tissues. apoptotic cell, red; DAPI, blue. **c** Immunohistochemistry analysis of Bcl-2 in mice kidney tissues. Scale bar = 50 μm. Results represent mean ± SEM for 6–8 mice. ***P < 0.001 vs NC. ^##^*P* < 0.01, ^###^*P* < 0.001 vs STZ. Abbreviations: *NC* normal control, *Wog* wogonin, *STZ* streptozotocin

By detecting the expression of apoptosis-related proteins, such as cleaved caspase-3, Bax, and Bcl-2, we discovered that podocyte apoptosis was increased in diabetic mice compared to non-diabetic controls and was significantly improved by wogonin treatment (Fig. 11a). Moreover, TUNEL staining showed that wogonin could reduce cell apoptosis (Fig. 11b). Results of immunohistochemistry showed that downregulated Bcl-2 protein expression was rescued by wogonin (Fig. 11c). In short, our data proved the beneficial effect of wogonin on the main symptoms of DKD.

## Discussion

The present study explored the renoprotective role of wogonin in mice podocyte cells. Our evaluation of clinical symptoms in diabetic mice at 12 weeks showed a significant reduction in proteinuria after treatment with wogonin; however, no significant reduction in blood glucose level was observed. Furthermore, kidney inflammation was gradually worsened in diabetic mice, and wogonin showed an anti-inflammatory effect in these mice at 12 weeks. We found that wogonin played a key role in alleviating cell apoptosis and promoting autophagy in DKD by targeting Bcl-2. Our experimental studies highlighted the close link between dysregulated Bcl-2 and HG-induced MPC5 cells injury. Although previous studies showed pharmacological effects of wogonin on inflammation, apoptosis, oxidative stress, and cell cycle regulation, the underlying mechanisms have not been fully elucidated [8]. Thus, our findings indicated that wogonin regulates the severity of DKD by alleviating glomerular injury, albuminuria, and cell death. The possible mechanism for wogonin action on podocytes may be via alleviation of HG-induced apoptosis activation and autophagy disorder through targeting Bcl-2.

Wogonin has a wide range of biological activities and affects the inflammatory response, apoptosis, oxidative stress, and other aspects [12, 13, 19]. Wogonin reportedly protected against cisplatin-induced acute kidney injury by targeting RIPK1-mediated necroptosis [8, 20]. It prevented inflammation and fibrosis in diabetic nephropathy by inhibiting the NF-κB and TGF-β1/Smad3 signaling pathways [11, 20, 21]. In the current study, we demonstrated the anti-inflammatory effect of wogonin in HG-treated MPC5 cells. Further study revealed that wogonin significantly inhibited inflammation in STZ-induced diabetic mice. In particular, wogonin decreased the levels of inflammatory cytokines, such as TNF-α, MCP-1, and IL-1β. DKD is considered to be a chronic inflammatory disease, in which the structure of the glomeruli and kidney tubules is altered by chronic microinflammation, leading to proteinuria [22, 23]. Moreover, the study revealed a hyperglycemic environment promotes the activation of NF-κB signaling pathway, upregulates the expression of TNF-α, MCP-1, and IL-1β, thereby contributing to the renal damage in type 1 diabetic mice [24]. Thus, specific blocking of inflammatory cytokines or the above signaling pathway may reduce renal inflammation response and cell damage in DKD mice, thus alleviating DKD.

Autophagy dysfunction is involved in podocyte injury, and autophagy reportedly is inhibited in HG environment [4, 15, 25]. Our investigation revealed that wogonin treatment alleviated STZ-induced autophagy dysfunction in podocytes. To our knowledge, this is the first research to demonstrate that wogonin promoted autophagy in STZ-induced diabetic mice. Autophagy is a cellular biological process in which lysosomes degrade cytoplasmic components to maintain cell homeostasis [6, 26–28]. Mammalian target of rapamycin AMP-activated protein kinase and silent information regulator 1 (Sirt1) has important regulatory effects in autophagy [14]. Our results also highlighted that p62, the key protein of autophagy, was significantly increased in renal tissue from diabetic nephropathy patients.

Furthermore, we discovered that wogonin reduces podocyte apoptosis in DKD. Apoptosis is a strictly controlled process of cell death, which is necessary for cell growth and body development [29]. The apoptotic pathway is involved in the development of many diseases including diabetic nephropathy [17, 30]. Bax and Bcl-2 are the major mediators of endogenous apoptosis and apoptosis are activated by Bax and inhibited by Bcl-2. Under normal conditions, autophagy and apoptosis maintain a dynamic balance to ensure the stability of the internal environment. However, the decrease of protective autophagy and the increase of apoptosis of podocytes may be one of the important causes of DKD renal injury [31, 32]. Interestingly, we found that wogonin significantly increased the protein level of Bcl-2 while did not affect its mRNA expression, which revealed that wogonin regulated Bcl-2 possibly by reducing the degradation of Bcl-2 rather than promoting its production.

To better understand the mechanism of wogonin in DKD, we used DS software to analyze its molecular target. The interaction between wogonin and Bcl-2 was simulated by computer-aid. A crucial element in assessing the development potential of drugs is target engagement, which is measured by CETSA at various stages of drug development. In the present study, CETSA confirmed that wogonin bound to Bcl-2 with high binding affinity. Furthermore, molecular docking was used to elucidate the most stable binding posture on Bcl-2 active sites with wogonin. Importantly, we found that Bcl-2 expression levels were significantly decreased in renal tissues of diabetic nephropathy patients. Thus, Bcl-2 may have an important role in DKD.

To further confirm the role of Bcl-2 in podocyte injury in DKD, we performed an IP assay to verify the role of Bcl-2 in the regulation of autophagy and apoptosis. Interestingly, after wogonin treatment, the binding of Beclin-1 to Bcl-2 decreased, while that of Bax, a pro-apoptotic effector protein, to Bcl-2 increased, indicating inhibition of apoptosis and promotion of autophagy [17]. Autophagy and apoptosis play important roles in the development and cellular homeostasis [32, 33]. They may be triggered by common upstream signals, resulting in combined autophagy and apoptosis, or may be mutually exclusive. Recent studies have shown possible molecular mechanisms of interaction between autophagy and apoptosis [32]. Bcl-2 is a recognized anti-apoptotic factor, which inhibits Beclin-1 mediated autophagy by binding to Beclin-1 [31, 34]. In fact, Bcl-2 downregulates autophagy by interacting with Beclin-1, the autophagy effector. Beclin-1 contains a BH3 domain similar to Bcl-2, which is required for binding to anti-apoptotic Bcl-2 and is also necessary for Bcl-2-mediated autophagy inhibition [33]. Moreover, Bax/Bak translocates to the mitochondrial membrane in response to apoptotic stimuli, promotes the release of cytochrome *c* into the cytosol from the mitochondrial membrane space, and the anti-apoptotic members, Bcl-2 can interact with Bax to inhibit apoptosis [17]. Previous research reported that phosphorylation of Bcl-2 on Ser^70^ could inhibit Bcl-2 degradation, stabilized Bcl-2–Bax interaction to inhibit apoptosis, and disrupted the Bcl-2–Beclin-1 complex to promote autophagy [18]. Thus, Bcl-2 represents a bridge between autophagy and apoptosis. Our data further found that Bcl-2 could be a potential target for wogonin through improving the disorder of autophagy and apoptosis of podocytes.

In conclusion, we demonstrated that the renoprotective effect of wogonin in STZ-induced diabetic mice was mediated by targeting Bcl-2-mediated apoptosis and autophagy. We found that wogonin, used as a single agent, was sufficient to significantly reduce the symptoms of DKD and attenuate podocyte injury in vivo. Therefore, wogonin might serve as a promising therapeutic agent for treating DKD. Moreover, further exploration of Bcl-2 analogs and other drugs based on the interaction between wogonin and Bcl-2 might help identify more effective and safer drugs, with potential for translation to the clinic, to treat patients with DKD.
